# The micronucleus test—most widely used *in vivo* genotoxicity test—

**DOI:** 10.1186/s41021-016-0044-x

**Published:** 2016-10-01

**Authors:** Makoto Hayashi

**Affiliations:** makoto international consulting (mic), Kami-imaizumi, Ebina, Kanagawa 243-0431 Japan

**Keywords:** Micronucleus, Historical consideration, Chromosomal aberration, *in vivo*, Animal welfare

## Abstract

Genotoxicity is commonly evaluated during the chemical safety assessment together with other toxicological endpoints. The micronucleus test is always included in many genotoxic test guidelines for long time in many classes of chemicals, e.g., pharmaceutical chemicals, agricultural chemicals, food additives. Although the trend of the safety assessment of chemicals faces to animal welfare and *in vitro* systems are more welcome than the *in vivo* systems, the *in vivo* test systems are paid more attention in the field of genotoxicity because of its weight of evidence. In this review, I will summarize the following points: 1) historical consideration of the test development, 2) characteristics of the test including advantages and limitations, 3) new approaches considering to the animal welfare.

## Background

The micronucleus was recognized in the end of the 19th century when Howell and Jolly found small inclusions in the blood taken from cats and rats. The small inclusions, called Howell-Jolly body, are also observed in the erythrocytes of peripheral blood from severe anemia patients. These are the first description of the micronucleus itself. In this review, I introduce historical aspects on the use of the micronucleus test as one of *in vivo *mammalian genotoxicity tests.

## Historical consideration of the micronucleus test

The micronucleus was recognized in the end of the 19th century when Howell and Jolly found small inclusions in the blood taken from cats and rats. The small inclusions, called Howell-Jolly body, are also observed in the erythrocytes of peripheral blood from severe anemia patients. These are the first description of the micronucleus itself.

In 1959, Evans et al. [1] reported that gamma-rays induced micronuclei in root tips of kidney beans, and tried to evaluate the chromosomal aberration quantitatively. This was the first report to evaluate chromosomal aberration by the frequency of cells harboring micronucleus among normal cells and they estimated that about 60 % of the chromosomal fragments contributed to micronucleus formation.

In 1970, Boller and Schmid [2] developed a test method to evaluate the frequency of micronucleated erythrocytes among normal erythrocytes, which lack their own nuclei during hematopoiesis, using bone marrow and peripheral blood cells of Chinese Hamster treated with a strong alkylating agent, trenimon. In the paper, they named this method as "Mikrokern-Test (micronucleus test)". Up to the mid 1970's Schmid [3] and Heddle's [4] group built up the basics of the micronucleus test.

In 1976, Countryman and Heddle [5] reported a method using human cultured lymphocytes. Modifications have been introduced by Fenech and Moley [6] using cytocharasin B and now the method is widely used for human monitoring.

In 1979, Cole et al. [7] and King and Wild [8] observed induction of micronucleus which appears in the fetal mouse liver and peripheral blood cells at the very late stage of gestation whose mothers were treated intraperitoneally with a clastogenic chemical. Some chemicals get metabolized in the liver and become their active form. When the active forms are unstable and diminish before reaching the bone marrow, the clastogenicity of these chemicals will not be detected by the usual method. Using fetal micronucleus method, however, the unstable active metabolites (e.g., dialkyl-nitrosamine) could be detected [9].

In 1980, MacGregor et al. [10] reported a method to detect micronucleus in mouse peripheral erythrocytes. Micronucleus is hardly seen in the peripheral blood of rats and humans because erythrocytes including micronuclei are captured and destroyed by the spleen rapidly and effectively. In mice, however, micronucleated erythrocytes exist just the same as normal cells in the peripheral blood. The assay using bone marrow evaluates an acute effect of chemicals but the method using mouse peripheral blood erythrocytes can evaluate a chronic effect of the test chemical by analyzing of mature erythrocytes which harboring micronuclei up to their life span.

In 1981 and 1983, Lähdetie et al. [11] and Tates et al. [12] reported a method using male germ cells. The reports suggested the possibility of using the micronucleus test to detect potential of heritable adverse effects of chemicals.

In 1983, the micronucleus committee of US-EPA Gene Tox Program reported an overall summary including database of evaluated micronucleus test results of chemicals assayed up to that time [13]. An international collaborating program to evaluate short term studies has also started by International Program on Chemical Safety (IPCS) this year. Carcinogens and non-carcinogens were evaluated by several *in vivo* test systems and the micronucleus test obtained the best score to estimate the carcinogenic potential of chemicals among assays studied [14].

In 1983, Hayashi et al. [15] and MacGregor et al. [16] introduced the fluorescence staining method. Hayashi et al. reported a fluorescent staining method using acridine orange to identify specifically micronuclei by yellowish green fluorescence emitted from DNA concomitantly to identify immature erythrocytes by red fluorescence emitted from RNA. MacGregor et al. reported an alternative fluorescent staining method to identify micronuclei and immature erythrocytes using Hoechst 33258 and pyronin Y. These DNA and RNA specific methods contributed to increase the accuracy of scoring micronucleated immature erythrocytes.

From early 1980's, some countries as well as international organizations started to make their own test guidelines for evaluating safety assessment of chemicals. The micronucleus test is included in the genotoxicity test battery. Under the circumstance, from 1984 up to now, the Collaborative Study Group for the Micronucleus Test under the Mammalian Mutagenicity Study Group, which is a sub-organization of Environmental Mutagen Society of Japan (CSGMT/JEMS-MMS), started to study on various factors which could affect the test results. Up to date, the followings have been studied and reported: the sex-related difference [17], the strain difference [18], the difference between intraperitoneal injection and oral gavage application [19], the effect of the number of treatments [20], the micronucleus test with peripheral blood using acridine orange supravital staining method [21], the aging of mice [22, 23], the performance of the test on chemicals categorized in IARC groups 1, 2a and 2b [24], the micronucleus using rat peripheral blood [25], the 28-days repeat treatment micronucleus test [26], and the test targeted other than erythropoietic tissues [27, 28]. These outcomes of the collaborative trials have given a lot of impacts and valuable contributions to standardize the test protocol and accordingly to the test guidelines, e.g., ICH S2(R1) and OECD TG 474 [29, 30].

Micronucleus test can also detect spindle poisons [31], and in that case, the micronuclei are larger than usual in size [32, 33]. In 1988, the kinetochore specific staining using antibody was introduced to detect chemicals which cause dis-function of mitotic apparatus [34]. Hayashi et al. reported the method to isolate micronuclei from peripheral blood and to distinguish between micronuclei with and without centromere [35].

As early as 1986, the automation for the analysis of micronucleated erythrocytes has been approached by flow cytometry [36–39] and also by image analyzer systems [40, 41]. Using the flow cytometry, the dose-response relation was studied with ionizing radiation until very low dose level [42]. These kind studies can be practically because the flow cytometer can analyze a large number of cells in a short period, accordingly the statistical power to detect micronucleus induction would increase.

In 1990, the committee of US-EPA Gene Tox Program published a revised edition of the 1983's report [13, 43]. This revision includes new data and ideas for a new protocol. From 1992, the movement of the international harmonization of genotoxicity test systems from the methodological view point. In December 1992, as the pre-meeting for the International Workshop on Standardization of Genotoxicity Test Procedures (IWGT), organizers and chairpersons met together in Tokyo, and extracted questionable points from each assay which should be discussed at the workshop. In 1993, the international workshop was taken place for two days at Melbourne attached to the 6th International Conference on Environmental Mutagens and selected points were discussed and reported [44]. After that time, the IWGT, as a rule, took place as a satellite meeting of each ICEM [45, 46].

Recently, the OECD TG 474, Mammalian Erythrocyte Micronucleus Test was updated [30]. For any purposes the micronucleus test data should be reliable and accurate. Thorough understandings on the mechanism of micronucleus formation, and the characteristics and limitations of the method, the experiment should be carefully done by the experienced and skillful persons, and thereby the satisfactory and reliable outcomes will be obtained.

## Characteristics of the test including advantages and limitations

**Fig. 1:**
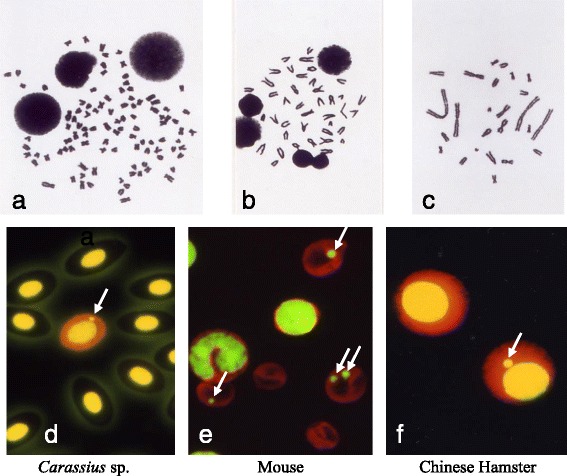
Comparison of the appearance between karyotypes and micronucleated cells. Karyotype and micronucleus (arrow) of fresh water fish (*Carassius* sp.) (**a** and **d**), mouse (**b** and **e**), and Chinese hamster (**c** and **f**). The micronuclei are simple structure independently from complexity of karyotype of each species

Micronucleus test does not take a direct observation of chromosomes in the cell metaphase. However, the micronucleus test is not suggested to be a simply alternative method for metaphase analysis or the results of the assay have less value compared to those of chromosomal aberration test. The author believes the micronucleus test as a related to but an independent method from chromosomal aberration test with its own characteristics. The characteristics of the micronucleus test compared to the metaphase analysis: <1 > any dividing cell population can be used regardless of its karyotype (Fig. 1), <2 > accurate data can be obtained because its endpoint is simple and easy to identify, <3 > response can be detected for longer duration, <4 > spindle poisons can also be detected, <5 > the background frequencies of micronucleated cells are usually stable, <6 > an additional treatment of chemicals other than test articles, e.g., colcemid or BrdU, is not required, but <7 > types of chromosomal aberration cannot be classified by micronuclei and <8 > possibility of appearance of pseudo-micronucleus under some circumstances.

All the issues are important advantages as well as limitation of the micronucleus test. Among these, “<2 > accurate data can be obtained because its endpoint is simple and easy to identify” to be discussed here. In the micronucleated erythrocyte, the micronucleus is only DNA component in the cell because the main nucleus of the cell is expelled during the erythropoiesis of mammals and the structure is simple. Such feature is suitable for identification of evidence of chromosomal aberration by machines automatically, e.g., image analysis or flow cytometry. A contemporary flow micronucleus test developed by Dertinger and his group is most widely used among automating scoring systems. Their system includes gating for young erythrocyte by immunostaining of erythrocyte membrane and also gating the amount of DNA. Usually, they analyze 20000 young erythrocytes but a million cells can be analyzed without any difficulty. This means the statistical power can be increased easily when the cell is considered to be a unit of analysis. The current revised OECD TG 474 proposed 4000 young erythrocytes should be analyzed, which is twice the number of cells stated in the former guideline. It is time consuming and tedious work to analyze 4000 young erythrocytes in the case of the ordinal microscopic analysis, but the flow cytometer can do that easily. The reproducibility of the outcomes between laboratories looks good and the system considered to be well validated.

Only the problem, however, is the interpretation of data that shows very slight increase compared to the control under some experimental conditions when the statistical power increased. It is not only the case of the micronucleus test but the other test systems with increase statistical power. For hazard identification it might be powerful but it is difficult to interpret in the case of the risk assessment. This is a difficult problem to be solved in the regulatory science [47, 48].

## New approaches considering to the animal welfare

Considering animal welfare, this *in vivo* test should approach to follow the 3Rs (Replacement, Reduction, and Refinement). One other hand, each test is required to show the high sensitivity, which means increment of test animals. To resolve such contradictive requirement, we have tried several approaches, i.e., 1) omission of the concurrent positive control group from the test protocol, 2) combination of two or more test systems, e.g., the micronucleus test and the comet assay, and 3) incorporation of the micronucleus test into other toxicological studies, e.g., repeat dose study.

1. The micronucleus test usually performed routinely to evaluate *in vivo* of chemicals. In such cases, the historical positive control can assure the performance of the test, and concurrent positive control group is not necessary to include in all tests. It is, however, recommended to use the positive control slides, which prepared separately, to certify the proper staining and observation. ICH S2(R1) guideline and OECD TG 474 accepted the omission of the concurrent positive control [29, 30, 49]. This approach reduces the number of test animals from each study.
2. Up to recently, the *in vivo* genotoxicity tests have been run standalone as other animal toxicological studies. To approach the 3Rs concept, two or more assay systems are combined as the multiple endpoint assays. The difficulty was how to overcome the different optimal treatment regime for each test, especially sampling time after the last treatment. Originally, this point was overcome by using peripheral blood as target cells of the micronucleus test and we do not have to kill animals at each sampling time, which adjust the protocol to the other assay system. For example, we can use transgenic animal model to detect gene mutations together with the micronucleus evaluation for chromosomal aberration. Recently, the combination of the micronucleus test and the comet assay is being used most frequently to detect together chromosomal aberration and DNA damage by 3 treatment protocol. Animals are killed at short period (e.g., 3 h) after the third treatment that fit optimal for the both assays [49].
3. The guideline of ICH S2(R1) recommends to integrate genotoxic endpoints, e.g., micronucleus formation, into general repeat dose toxicological study. Theoretically, we can take peripheral blood sample at any time during the study, and bone marrow cells at the termination of the study. Of course, there are several restrictions to perform the toxicological study completely, but we can use animals of the satellite group, if any. The most important point of the integration study is the dose levels. Generally, the dose levels of the repeat dose study are lower than those of the standalone or combination micronucleus test. The ICH guideline suggests when mammalian cell assay gives positive or omits mammalian assay, several factors should be evaluated to determine whether the top dose is high enough for the appropriate genotoxicity evaluation. If one or more criteria listed below are considered to be sufficient to evaluate *in vivo*:
  i. Maximum Feasible Dose (MFD) based on physico-chemical properties of the drug in the vehicle is similar to that achievable with acute administration.
  ii. Limit dose of 1000 mg/kg for studies of 14 days or longer, if this is tolerated.
  iii. Maximal possible exposure demonstrated either by reaching a plateau/saturation in exposure or by compound accumulation. In contrast, substantial reduction in exposure to parent drug with time (e.g., ≥ 50 % reduction from initial exposure) can disqualify the study (unless a blood sample taken in the first few days is available). If this is observed in one sex, generally the sex with reduced exposure would not be scored at the end of the study, unless there is enhanced exposure to a metabolite of interest.
  iv. Top dose is ≥ 50 % of the top dose that would be used for acute administration, i.e., close to the minimum lethal dose, if such acute data are available for other reasons. (The top dose for acute administration micronucleus tests is currently described in OECD guidance as the dose above which lethality would be expected; similar guidance is given (e.g., Hartmann et al., 2003) for other *in vivo* assays.)

Selection of a top dose based on only an exposure margin (multiple over clinical exposure) without toxicity is not considered to be sufficient justification. These approaches are appropriate for reduction of test animals and we should consider any of these approaches for the good practice of animal welfare.

## Conclusion

The *in vivo* rodent micronucleus assay has limitations as other genotoxicity assays, namely no one assay can detect all genotoxic chemicals with different modes of actions. It is, however, the micronucleus assay has been most widely used as an *in vivo* assay as the most reliable assay to assess the induction of chromosomal aberrations, which is one of two major endpoints of mutagenicity, for not only hazard identification but also risk assessment. There is also no doubt the assay has more weight in the course of risk assessment than other assays including *in vitro* mammalian chromosomal aberrations assay although it has shortcomings in the view point of animal welfare. At the present time, still animal studies are important for safety assessment of chemicals and the attempts mentioned in this review should be appreciated to reduce experimental animals. We have to make balance also in the field of safety assessment between weight of evidence and animal welfare.
